# An examination of the relationships between attention/deficit hyperactivity disorder symptoms and functional connectivity over time

**DOI:** 10.1038/s41386-021-00958-y

**Published:** 2021-02-08

**Authors:** Luke J. Norman, Gustavo Sudre, Marine Bouyssi-Kobar, Wendy Sharp, Philip Shaw

**Affiliations:** 1 Section on Neurobehavioral and Clinical Research, Social and Behavioral Research Branch, National Human Genome Research Institute, National Institutes of Health, Bethesda, MD USA; 2 Office of the Clinical Director, National Institute of Mental Health, National Institutes of Health, Bethesda, MD USA

**Keywords:** Attention, Developmental disorders

## Abstract

Previous cross-sectional work has demonstrated resting-state connectivity abnormalities in children and adolescents with attention/deficit hyperactivity disorder (ADHD) relative to typically developing controls. However, it is unclear to what extent these neural abnormalities confer risk for later symptoms of the disorder, or represent the downstream effects of symptoms on functional connectivity. Here, we studied 167 children and adolescents (mean age at baseline = 10.74 years (SD = 2.54); mean age at follow-up = 13.3 years (SD = 2.48); 56 females) with varying levels of ADHD symptoms, all of whom underwent resting-state functional magnetic resonance imaging and ADHD symptom assessments on two occasions during development. Resting-state functional connectivity was quantified using eigenvector centrality mapping. Using voxelwise cross-lag modeling, we found that less connectivity at baseline within right inferior frontal gyrus was associated with more follow-up symptoms of inattention (significant at an uncorrected cluster-forming threshold of *p* ≤ 0.001 and a cluster-level familywise error corrected threshold of *p* < 0.05). Findings suggest that previously reported cross-sectional abnormalities in functional connectivity within inferior frontal gyrus in patients with ADHD may represent a longitudinal risk factor for the disorder, in line with efforts to target this region with novel therapeutic methods.

## Introduction

Attention deficit/hyperactivity disorder (ADHD) is a childhood-onset neurodevelopmental disorder characterized by symptoms of age-inappropriate inattention and hyperactivity/impulsivity [1]. It affects around 5–10% of children and has a significant negative impact on mental health, family functioning, and educational attainment [1–4]. Distinct developmental courses have been described for inattention and hyperactivity/impulsivity symptom dimensions; inattention typically increases during early childhood, and then shows small declines or remains stable during adolescence, whereas hyperactivity/impulsivity tends to follow a sharp linear decline with age beginning in late childhood/early adolescence [5, 6]. However, particularly in the case of inattentive symptoms, substantial variation in symptom changes over the course of development have been observed. Some individuals show a steady decline in symptoms with age, whereas symptoms persist or increase for other individuals, and the mechanisms underlying these individual differences are poorly understood [4, 5].

While the exact pathophysiology of the disorder is unknown, the notion that ADHD is a disorder of dysfunctional brain connectivity has been hypothesized widely [7–10]. Studies using functional magnetic resonance imaging (fMRI) have pointed to abnormal connectivity within and between regions of ventral attention, inferior-fronto-striato-thalamic, fronto-parietal, and default mode networks in youths and adults with ADHD compared to unaffected individuals [11–18]. Current models often assume that these functional brain abnormalities underlie ADHD symptoms, and form part of the mechanistic pathway between genetic and environmental risk and symptoms of the disorder [19–21]. However, existing work has used primarily cross-sectional case–control designs, and it is also possible that functional brain abnormalities are secondary consequences of ADHD symptoms [4, 22, 23]. Distinguishing brain abnormalities that play etiological roles in the onset, maintenance, or worsening of ADHD symptoms from those that are secondary consequences or epiphenomenal correlates of the disorder is important in light of ongoing efforts to find potential targets for novel, biomarker-driven interventions in ADHD [24].

**Fig. 1:**
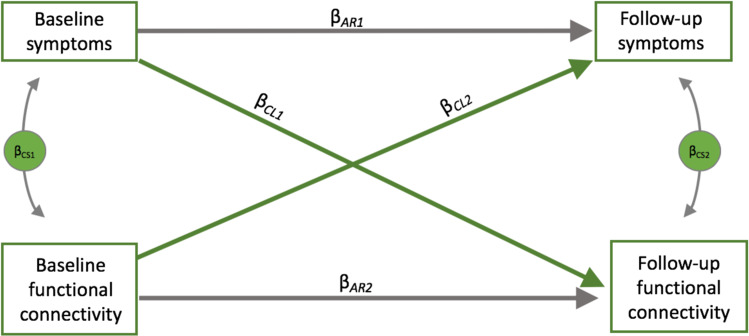
Depicts the general modeling strategy used. The first cross-lagged coefficient *β*_CL1_ represents the association between ADHD symptoms assessed at baseline and functional connectivity measured at follow-up that have been adjusted for functional connectivity measured at baseline. The cross-lag coefficient, *β*_CL2_, represents the association between functional connectivity measured at baseline and ADHD symptoms measured at follow-up that have been adjusted for baseline ADHD symptoms. Cross-sectional associations between functional connectivity and ADHD symptoms were also modeled (*β*_CS1_ and *β*_CS2_). Autoregressive coefficients represent the stability of ADHD problems (*β*_AR1_) and functional connectivity (*β*_AR2_) from baseline to follow-up. Covariates of no interest including mean study age, sex, and length of time between scans were also controlled for in the model (not shown).

In this work, we examined the relationships between functional connectivity and ADHD symptoms over time using voxelwise autoregressive cross-lagged modeling. Cross-lag models are rarely used in neuroimaging studies, but are a variant of structural equation models which allow for potentially reciprocal longitudinal relationships between two variables to be modeled concurrently [25]. An overview of the general modeling approach is given in Fig. 1. We were interested in examining the relationship between baseline functional connectivity and follow-up symptoms as well as the relationship between baseline symptoms and follow-up functional connectivity, while also modeling contemporaneous associations and the stability of each variable over time.

We characterized functional connectivity using a whole-brain graph–theoretical measure of global brain connectivity (eigenvector centrality mapping, ECM) [26, 27]. ECM was adopted due to its exploratory, data-driven approach which does not require a priori defined seed regions or network parcellations. Voxelwise global brain connectivity methods, including ECM, have been used successfully elsewhere to study age-related changes in connectivity in healthy youth and adults [28–31], to study differences in connectivity between psychiatric and neurodevelopmental disorder patients and healthy controls [11, 28, 32, 33], and in neuropharmacological research [34–36]. An advantage over other global brain connectivity methods is that ECM takes into account not only the strength of a voxel’s direct connections but also whether or not those connections are to voxels that are highly connected in the network. That is, the eigenvector centrality of a voxel is high if it is connected to prominent voxels which themselves have high centrality [26, 27].

Accumulating research findings are most consistent with ADHD existing at the extreme end of a continuum of ADHD traits, which are also present in the general population [37–39]. This extends to the brain, with previous work showing an overlap in brain regions differing between patients with ADHD and controls with those correlated with ADHD traits in the general population [37, 38, 40]. Consequently, in this work, we were interested in the relationship between ADHD symptoms and brain functioning over time, regardless of diagnostic status, with this approach allowing us to include subjects who were transitioning between diagnostic categories (e.g., from asymptomatic to clinical, or clinical to subclinical) during development.

Based on previous cross-sectional work, we hypothesized that the cross-lag analyses would demonstrate longitudinal associations between ADHD symptoms and the connectivity of ventral attention, inferior-fronto-striato-thalamic, fronto-parietal, and default mode network regions [13, 16, 18, 41]. However, given that this is the first cross-lag study to examine this research question, we did not form specific hypotheses regarding the direction of effects.

## Materials and methods

### Participants

We included youths aged 5–18 years old selected from ongoing accelerated longitudinal cohort studies at the National Institutes of Health [14, 42, 43]. For inclusion, participants had to have two good quality resting-state scans at least 0.5 years apart and contemporaneous symptom assessments. Given our interest in ADHD, the sample was enriched for ADHD symptoms. This was achieved, in part, by targeting the recruitment of children and adolescents with ADHD traits regardless of diagnostic status, in addition to the specific recruitment of children and adolescents with ADHD diagnoses (e.g., from local clinics and support groups). We also included data from our study of multigenerational ADHD families, in which multiple family members met diagnosis for ADHD [14]. Therefore, across the entire dataset analyzed for the present analysis, subjects were from 167 nuclear families. Of these, 120 had one member and 47 had more than one member within our age range and with usable imaging data and concurrent symptom assessments. Owing to the non-independence of observations, we included only one subject per nuclear family in the analysis. Given our interest in the relationship between brain connectivity and ADHD symptoms, when selecting subjects for inclusion from each family we chose the family member reporting >0 symptoms during at least one time point. Where all subjects from a family reported either zero symptoms or greater than zero symptoms, subjects were chosen based on image quality (i.e. smallest amount of motion, averaged over the two scans). The results also remained when choosing all subjects based on image quality. See Supplement for details.

ADHD symptoms were assessed using the parental Diagnostic Interview for Children and Adolescents-IV (DICA-IV) which establishes the number of symptoms of inattention and hyperactivity/impulsivity (with a range from 0 to a maximum of 9 symptoms in each category) [44]. Interviews were conducted by two experienced clinicians (W.S. and P.S.) with interrater reliabilities of *κ* > 0.9. If subjects were taking psychostimulant medication, this was withdrawn at least 24 h before testing, and parents rated symptoms as they appear while the child is off medication. Potential comorbid disorders were also determined via clinical interviews. Exclusion criteria included IQ less than 80 as determined via the Wechsler intelligence scale [45, 46], neurologic disorders affecting brain structure, current substance dependence, or psychotic disorders. Other disorders, including mood, anxiety, autism spectrum, and oppositional defiant disorders, were permitted. Written informed consent and assent was obtained for all patients and/or their legal guardians, according to procedures reviewed and approved by the institutional review board of the National Human Genome Research Institute, who also approved the research protocol.

### fMRI acquisition and preprocessing

Resting-state fMRI was acquired using a gradient-echo-planar series [repetition time = 2500 ms; echo time = 27 ms; flip angle = 90°; 44 axial contiguous interleaved slices per volume; 2.8-mm slice thickness; field of view = 22 cm; 64 × 64 acquisition matrix; single-voxel volume = 3.4, 3.4, 2.8 mm] with whole-brain coverage on a 3-T GE scanner for 315 s (General Electric). Participants were instructed to lie in the scanner at rest, looking at a fixation cross. A baseline-weighted anatomical image [magnetization prepared rapid acquisition gradient recalled echo sequence (MP RAGE)]: 124 axial slices, 1.3-mm slice thickness, field of view = 22 cm, 224 × 224 acquisition matrix] was acquired to assist with the alignment of the functional image with normalization to stereotaxic space.

Preprocessing was performed using a validated and standardized 36-parameter plus despiking pipeline [47–51], implemented in fMRIPrep and xcpEngine software packages [47, 52]. Full details are given in the Supplement. Following this, functional connectivity was assessed using ECM, a form of voxelwise global brain connectivity mapping based on graph theory principles, as implemented in the fastECM package (github.com/amwink/bias/tree/master/matlab/fastECM) [11, 26]. As compared to traditional ECM calculation methods, the fastECM algorithm is more computationally efficient because it computes matrix–vector products without having to compute or store the entire connectivity matrix [27]. As the matrix for fastECM computation must be symmetric and positive, we adopted the rectified linear unit correlation method as described in detail elsewhere to guarantee an entirely positive correlation matrix [53]. To facilitate second-level analyses, resulting ECM maps were transformed to a Gaussian normal distribution and residualized for motion [34].

### Statistical analysis

#### Descriptive analyses

 Wilcoxon signed-rank tests examined within-subject changes in symptoms from baseline to follow-up.

#### Cross-lagged analyses

Voxelwise autoregressive cross-lagged path models were implemented in the Lavaan package [25] for R (http://www.r-project.org). We were interested in determining, at each voxel, the relationship between baseline functional connectivity and follow-up symptoms while controlling for baseline symptoms. Conversely, we also estimated the relationship between baseline symptoms and follow-up brain connectivity while controlling for baseline connectivity. The model also controlled for contemporaneous associations between these two variables and the stability of each variable across timepoints. Sex, medication status (linear term: 0 = unmedicated, 1 = medicated at one time point, 2 = medicated at both timepoints), each subject’s age averaged across timepoints and the length of time between baseline and follow-up scans for each subject were included as covariates of no interest.

A significant proportion of subjects had zero symptoms at both timepoints (22.16% for inattention symptoms and 31.74% for hyperactivity/impulsivity symptoms). Therefore, follow-up robustness checks were performed (i) excluding subjects who had zero symptoms at both timepoints and (ii) using zero-inflated Poisson regression. The latter model allowed us to examine whether regions from the primary voxelwise analysis survived after controlling for zero inflations, and were performed using the pscl package for R [54, 55]. See Supplement. Owing to their distinct developmental trajectories, inattention and hyperactivity/impulsivity symptoms were modeled separately [5].

**Fig. 2:**
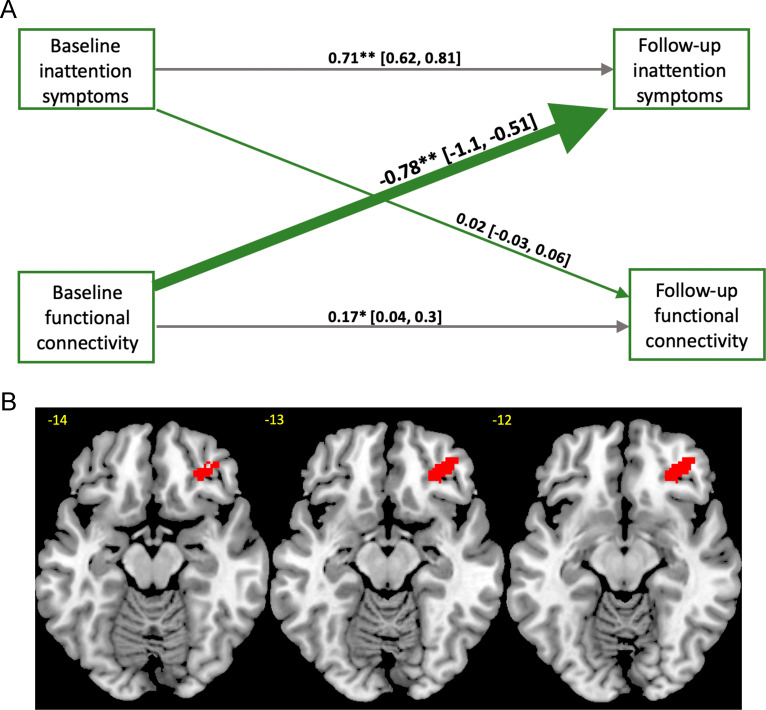
Results from the voxelwise cross-lag model. Panel **A** depicts the cross-lagged panel model in which baseline connectivity within right inferior frontal gyrus was associated with follow-up symptoms of inattention. Numeric values for the cross-lag estimates are standardized structural regression coefficients and are presented along with their 95% confidence intervals. Standardized autoregressive coeffecients on the horizontal lines represent the stability of symptoms and right inferior frontal gyrus connectivity over time. **B** Axial brain slices showing the significant inferior frontal gyrus cluster. Brain images were thresholded at an uncorrected cluster-forming threshold of *p* ≤ 0.001 and an FWE cluster corrected threshold of *p* < 0.05/4 = 0.0125. *<0.05, **<0.001.

Voxelwise maps for the cross-lagged associations between symptoms and functional connectivity were thresholded at an initial uncorrected cluster-forming threshold of *p* ≤ 0.001 and then at a familywise error (FWE) corrected cluster threshold of *p* < 0.05/4 = 0.0125, using Gaussian random field theory [56]. The cluster threshold of *p* < 0.0125 was chosen to correct the *α* = 0.05 for the four contrasts of interest (connectivity to inattention symptoms, inattention symptoms to connectivity, connectivity to hyperactivity/impulsivity symptoms, hyperactivity/impulsivity symptoms to connectivity) using Bonferroni correction. Eklund and colleagues recommended the use of a relatively conservative cluster-forming threshold of *p* ≤ 0.001 along with an FWE corrected cluster threshold of *p* < 0.05, as they showed that it adequately controls the false-positive rate in group-level fMRI analyses [57]. In order to visualize and summarize the results, and to perform sensitivity analyses and robustness checks, mean ECM values for clusters that were significant in the voxelwise cross-lagged analysis were extracted from each subject’s Gaussian-normalized and motion-residualized images using the 3dmaskave function in AFNI [58], and entered into cross-lag models. Values from these follow-up models were included in Fig. 2. To further check the robustness of findings, we repeated analyses based on extracted cluster values after removing subjects with the lowest and highest 15% of between-scan durations. Data will be shared on individuals who provided consent in accordance with NIH policy, and all code is available on request to the corresponding author.

## Results

### Sample demographics and descriptive analyses

One hundred and sixty-seven children and adolescents had functional connectivity data acquired at a mean interval of 2.56 years (SD = 1.5, range = 0.75–6.84 years) in tandem with ADHD symptom assessments. A total of 88 (52.69%) subjects met DSM-5 diagnostic criteria for ADHD during at least one time point, defined by the presence of six or more impairing symptoms of inattention, six or more symptoms of hyperactivity/impulsivity, or both [1]. Of these, 57 met criteria at both time points, 22 at baseline only, and 9 at follow-up only. In addition, 19 subjects met diagnosis for one or more other psychiatric disorders at time 1, while 23 met diagnosis for one or more additional disorders at time 2 (see Table 1). Demographic details, symptom scores, and medication statuses for subjects are given in Table 1. Wilcoxon signed ranks tests showed that while hyperactivity/impulsivity symptoms decreased significantly from baseline to follow-up (*V* = 3713, *p* < 0.001), a similar decrease did not reach significance for inattention symptoms (*V* = 2877, *p* = 0.069). However, as can be observed from Supplementary Figs. 1 and 2, there was also substantial variation between individuals in how symptoms changed over time, with some individuals showing steady declines in symptoms with age, whereas other individuals showed increasing or persisting symptoms.

**Table 1:** Subject demographic and clinical characteristics (*N* = 167).

	Time 1		Time 2	
	*N*	%	*N*	%
Sex—female	56	33.53	–	–
Meets ADHD diagnosis	79	47.31	66	39.52
Stimulant medication	59	35.33	66	39.52
**Race/ethnicity**				
White (non-Hispanic)	119	71.26	–	–
White (Hispanic)	10	5.99	–	–
Black	19	11.38	–	–
Asian	6	3.59	–	–
Other/mixed	13	7.78	–	–
**Comorbidities**				
Anxiety disorders	6	3.6	10	6
Mood disorders	2	1.98	5	2.99
Oppositional defiant disorder	11	6.59	8	4.79
Autism spectrum disorder	2	1.2	4	2.4
	Mean	SD	Mean	SD
Age (years)	10.74	2.54	13.3	2.48
Motion (mean RMS)	0.2	0.14	0.15	0.1
IQ	111.48	14.88	–	–
Hollingshead Scale (SES)	33.83	16.29	–	–

*ADHD* Attention deficit/hyperactivity disorder, *IQ* intelligence quotient, *RMS* root‐mean‐square, *SES* socioeconomic status.

### Voxelwise cross-lagged analysis

In the voxelwise cross-lagged analysis, we found that baseline connectivity within right inferior frontal gyrus was associated negatively with inattention symptoms at follow-up (Montreal Neurological Institute, MNI *x*, *y*, *z* = 38, 38, −8, Max-*Z* = −4.49, *k* = 150; *B* = −0.78, SE = 0.15, *z* = −5.16, *p* < 0.0001). This cluster was also observed after removing subjects with zero symptoms at both timepoints, albeit at a slightly relaxed cluster-forming threshold of *p* ≤ 0.005 (MNI *x*, *y*, *z* = 32, 34, −10, Max-*Z* = −4.39, *k* = 201; *B* = −0.93, SE = 0.22, *z* = −4.73, *p* < 0.0001). In other words, subjects with relatively less connectivity within this region at baseline had more symptoms of inattention at follow-up. See Fig. 2.

Autoregressive coefficients indicated that inattention scores measured at baseline were associated positively with follow-up symptom scores, suggesting stability in this measure in addition to some interindividual variability over time (*B* = 0.71, SE = 0.05, *z* = 14.52, *p* < 0.0001). The same was true for connectivity of the right inferior frontal gyrus (*B* = 0.17, SE = 0.07, *z* = 2.49, *p* = 0.01). There were no significant findings for hyperactivity/impulsivity symptoms.

### Sensitivity analyses and robustness checks

Further sensitivity analyses and robustness checks were based on extracted mean ECM values from clusters that were significant in the primary voxelwise analysis. Findings remained in a subsample of *N* = 117 who had a narrower range of between-scan intervals created by removing subjects who had intervals falling into the top 15% shortest or top 15% longest durations (the lower interscan interval becoming 1.13 and the upper 3.95 years, with mean = 2.31 years, SD = 0.86). Although the logistic model predicting excessive zeroes in time 2 inattention symptom scores using time 1 inferior frontal gyrus connectivity was significant (*B* = −2.7, SE = 1.01, *z* = −2.65, *p* = 0.008), our primary findings also survived after controlling for zero inflations using zero-inflated Poisson regression (*B* = −0.12, SE = 0.05, *z* = −2.26, *p* = 0.02).

## Discussion

The current study sought to clarify the relationships over time between symptoms of ADHD and functional brain connectivity. We observed a large degree of interindividual variability in symptom change with age, which furthermore was associated with individual differences over time in the functional connectivity of an inferior frontal region commonly implicated in the disorder [19, 20, 24, 59]. Specifically, greater connectivity of right inferior frontal gyrus at baseline was associated with fewer inattention symptoms at follow-up.

The right inferior frontal gyrus is a pivotal component of ventral attention and inferior-fronto-striato-thalamic networks that underlie attention and inhibitory control [20, 24, 60]. Reduced functioning within the inferior frontal gyrus is arguably the most consistent neuroimaging finding in ADHD [19, 20, 24], and a large body of research has reported reduced connectivity between inferior frontal gyrus and striato-thalamic, medial frontal and temporo-parietal regions in individuals with the disorder relative to controls [12, 13, 17, 18, 41, 61–64]. Previous work in ADHD has also reported dysfunction in the right inferior frontal gyrus during attention allocation, sustained attention, and inhibitory control [12, 17, 19, 20, 59, 61, 65–69], with this deficit in brain function argued to underlie the impairments in these cognitive domains that are observed as part of the disorder [19, 20, 70].

We extend prior work by reporting on the direction of effects between dysfunction in this brain region and symptoms of ADHD over time. Specifically, relatively lower connectivity at baseline within this region was a longitudinal risk factor for having more inattention symptoms at follow-up. This finding supports a potential mechanistic role for inferior frontal gyrus hypoconnectivity in the worsening and/or maintenance of inattention symptoms [19, 20, 24]. Finding that greater connectivity within right inferior frontal gyrus was associated at a later time point with better symptom outcomes is consistent with reports that stimulant medications known to improve ADHD symptoms may do so via a normalization of inferior frontal gyrus hypoactivation and hypoconnectivity [20, 71], and supports the rationale behind efforts to treat ADHD by targeting inferior frontal gyrus functioning directly using neurofeedback and brain stimulation methods [72, 73]. Previous work has also reported that adults with remitted childhood ADHD show greater inferior frontal gyrus functioning compared to those with persistent ADHD symptoms, a finding which has been interpreted as suggesting a normalization of inferior frontal gyrus functioning with time as symptoms remit [65, 74]. Intriguingly, the present findings suggest the potential for a different relationship; those with relatively preserved inferior frontal gyrus functioning may be better placed to go on to remit from ADHD [75].

No cross-lag findings were observed for hyperactivity/impulsivity symptoms, unlike for inattention symptoms. This might suggest that cross-lagged relationships between functional connectivity as assessed using ECM and ADHD symptoms are not present for hyperactivity/impulsivity symptoms. However, these symptoms were lower overall compared with inattention symptoms in the present sample and also showed a decline between timepoints for most subjects reporting symptoms at baseline. Therefore, future work focused on earlier developmental periods characterized by higher hyperactivity/impulsivity symptoms may delineate longitudinal neuroimaging biomarkers for this symptom domain [5].

Limitations of the study include the heterogeneity in age and interscan durations owing to our use of data from an accelerated longitudinal cohort study, although these variables were controlled for in the statistical model. Additionally, the findings survived in a sensitivity analysis based on a sub-group of subjects who had follow-up scans within a narrower range of 1.05–3.4 years following the first scan. A second limitation is that a number of subjects were prescribed stimulant medications during the course of the study. However, scans and symptom ratings were performed off psychostimulant medication and the model controlled for medication status. A third limitation is that we included only two timepoints, which meant that we could only evaluate linear changes. This work should therefore be considered “preliminary”, and followed up using datasets with enough data collected at ≥3 timepoints. Fourth, the mean age at the first time point was 10.74 years old, meaning that for many subjects we were only able to study longitudinal neural associations with symptom improvement, maintenance, or worsening, rather than associations with symptom onset. Fifth, a number of subjects reported zero symptoms at both timepoints, although findings survived when including only subjects who reported at least one symptom and after controlling for zero-inflation. In future work, it will be beneficial to collect longitudinal ADHD assessments using measures such as the strengths and weaknesses of ADHD symptoms and Normal behavior scale which provide a more continuous and normally distributed assessment of ADHD traits in the general population [76]. Finally, while ECM has numerous advantages including the lack of a requirement for a priori seeds or network definitions, as a summary measure of each voxel’s global connectivity to the rest of the brain it may not be sensitive to more localized connectivity abnormalities between specific sets of regions or networks [11].

In summary, we extend previous cross-sectional neuroimaging studies by showing that hypoconnectivity of the right inferior frontal gyrus preceded a worsening of inattentive symptoms, a finding that is in line with a proposed etiological significance for inferior frontal dysfunction as a longitudinal and mechanistic risk factor for ADHD [19, 20, 24, 73].

## Funding and disclosure

The study was funded by the Intramural Programs of the National Institute of Mental Health and National Human Genome Research Institute (Z01-HG200378). The authors report no competing financial interests in relation to the work described.

## Supplementary information

SUPPLEMENTAL MATERIAL
