# Supplementary material for: An examination of the relationships between attention/deficit hyperactivity disorder symptoms and functional connectivity over time
Source: Neuropsychopharmacology. 2021 Feb 8;47(3):704–10. doi: 10.1038/s41386-021-00958-y (PMC8782893; doi:10.1038/s41386-021-00958-y)
Supplement: Supplementary file 1 — SUPPLEMENTAL MATERIAL [file 41386_2021_958_MOESM1_ESM.docx]

**Supplement**

**Preprocessing of fMRI images**

Results included in this manuscript come from preprocessing performed using fMRIPrep version 1.3.2, a Nipype based tool [52,77]. Each T1w (T1-weighted) volume was corrected for intensity non-uniformity using N4BiasFieldCorrection v2.1.0 [78] and skull-stripped using antsBrainExtraction.sh v2.1.0 (using the OASIS template). Spatial normalization to MNI space was performed through nonlinear registration with the antsRegistration tool of ANTs v2.1.0 [79], using brain-extracted versions of both T1w volume and template. Brain tissue segmentation of cerebrospinal fluid (CSF), white-matter (WM) and gray-matter (GM) was performed on the brain-extracted T1w using fast (FSL v5.0.9) [80].

Functional data were slice time corrected using 3dTshift from AFNI v16.2.07 [58] and motion corrected using mcflirt (FSL v5.0.9) [81]. This was followed by co-registration to the corresponding T1w using boundary-based registration [82] with six degrees of freedom, using flirt (FSL). Motion correcting transformations, BOLD-to-T1w transformation and T1w-to-template (MNI) warp were concatenated and applied in a single step using antsApplyTransforms (ANTs v2.1.0) using Lanczos interpolation.

The final rsFMRI preprocessing steps used the xcpengine toolbox version 1.0 (https://xcpengine.readthedocs.io), and the 36-parameter + despiking functional design for deconvolution [47–51]. Despiking refers to the removal of and interpolation over intensity outliers in each voxel’s time series using AFNI’s 3DDESPIKE utility [58]. We then performed demeaning and removal of any linear or quadratic trends. The 36 parameters regressed from the timeseries included the 6 motion estimates, global signal, 2 physiological time series (white matter and cerebrospinal fluid), and then their derivatives, quadratic terms, and squares of derivatives [47,51]. Temporal filtering was performed using a bandpass filter of 0.01–0.08 Hz (first-order Butterworth filter [83]) and images were smoothed in SUSAN using a Gaussian-weighted kernel with 6 mm FWHM [84].

Eigenvector centrality was examined within a gray-matter mask generated by including voxels with a probability >=0.2 in the SPM8 (Wellcome Functional Imaging Laboratory, London, England) gray matter template and excluding voxels that fell on cerebellar regions defined by the Automated Anatomical Labeling template [85], or which had coverage in less than 95% of scans. For second-level analyses, the search space included frontal, temporal and parietal lobes as well as subcortical structures but excluded occipital lobe in addition to the cerebellum due to a lack of *a priori* interest and poor cerebellum coverage in some scans collected as part of our cohort.

All raw images underwent visual inspection. Quality control procedures also involved inspecting visual reports produced by fMRIPrep for brain extraction, segmentation, EPI to T1 co-registration, co-registration to standard space and the placement of white matter and cerebrospinal masks. Carpet plots summarizing the time series for all voxels within the brain mask were also checked [86]. Only scans with a mean relative root-mean-square displacement (mean-RMS) ≤0.5 were included in this analysis [81], a threshold used elsewhere in developmental research [87].

Three-hundred and ten subjects fully completed two resting-state scans at least 0.5 years apart along with concurrent symptom assessments. Of these, one subject was removed due to lacking a usable anatomical scan required for preprocessing. Two subjects had a functional scan that failed to complete the preprocessing pipeline. One functional scan for one subject had severe artifacts that were observed during visual checks. Data for a total of 306 subjects with two available scans completed preprocessing. From these, 86 subjects were removed from the analysis due to gross excessive motion in at least one of the scans [87], meaning that they had <2 scans available for inclusion. This left a sample of N=220 from 167 families. Due to the non-independence of observations, we included only one member per nuclear family. When selecting subjects for inclusion from each family, we chose the family member reporting >0 symptoms during at least one timepoint. Where all subjects from a family reported either zero symptoms or greater than zero symptoms, subjects were chosen based on image quality (i.e. smallest amount of motion, averaged over the two scans). This led to a final sample of N=167. Details on included and excluded scans are given in Supplementary Table 1.

**zero-inflated Poisson model**

A robustness analysis involving a zero-inflated Poisson model was run in the pscl package for R [54,55]. Such models have two sub-models: (i) a Poisson sub-model and (ii) a logit sub-model for predicting excess zeros. We were interested to examine whether the relationship between time one connectivity in the IFG (IFG_t1) and time two symptoms of inattention (symptoms_t2) survived after controlling for excess zeros (highlighted).

Poisson model

symptoms_t2 ~ symptoms_t1 + **IFG_t1** + IFG_t2 + gender + medication_status + duration_between_scans + mean_age

Logit model

symptoms_t2 ~ symptoms_t1 + IFG_t1 + IFG_t2 + gender + medication_status + duration_between_scans + mean_age

|  | **Included**  **(N=167)** | | **Excluded**  **(N=143)** | | **Statistical analysis** |
| --- | --- | --- | --- | --- | --- |
|  | **N** | **%** | **N** | **%** |  |
| **Sex - female** | 56 | 33.53 | 53 | 37.06 | χ2=0.42, p=0.5 |
| **Meets ADHD diagnosis** | 88 | 52.6% | 63 | 44.05 | χ2=2.3, p=0.13 |
| **Stimulant medication** | 80 | 47.9 | 41 | 28.67 | χ2=11.97, p<0.001 |
| **Race/ethnicity** |  |  |  |  |  |
| White (non-Hispanic) | 119 | 71.25 | 90 | 62.94 | χ2=2.43, p=0.12 |
| White (Hispanic) | 10 | 5.99 | 7 | 4.9 | χ2=0.18, p=0.67 |
| Black | 19 | 11.38 | 15 | 10.49 | χ2=0.06, p=0.8 |
| Asian | 6 | 3.59 | 9 | 6.29 | χ2=1.2, p=0.27 |
| Other/mixed | 13 | 7.78 | 22 | 15.38 | χ2=4.44, p=0.04 |
| **Comorbidities** |  |  |  |  |  |
| Anxiety disorders^1^ | 14 | 8.98 | 8 | 5.59 | χ2=0.9, p=0.34 |
| Mood disorders | 7 | 4.19 | 2 | 1.4 | χ2=2.13, p=0.14 |
| Oppositional defiant disorder | 16 | 9.58 | 14 | 9.79 | χ2=0.004, p=0.95 |
| Autism spectrum disorder^2^ | 4 | 2.4 | 1 | 0.7 | χ2=1.4, p=0.24 |
|  | **Mean** | **SD** | **Mean** | **SD** |  |
| **Age (years)** | 12.02 | 2.81 | 10.63 | 2.9 | t(618)=6.05, p<0.001 |
| **Motion (mean-RMS)** | 0.18 | 0.12 | 0.69 | 1.01 | t(618)=-9.15, p<0.001 |
| **Inattention symptoms** | 4.05 | 3.18 | 3.16 | 3.34 | t(618)=3.39, p<0.001 |
| **Hyperactivity/impulsivity symptoms** | 2.33 | 2.78 | 2.02 | 2.83 | t(618)=1.37, p=0.17 |
| **IQ** | 111.48 | 14.88 | 104.05 | 14.74 | t(618)=6.22, p<0.001 |
| **Hollingshead Scale**  **(SES)** | 33.83 | 16.29 | 33.88 | 17.17 | t(618) -0.04, p=0.97 |

**Supplementary Table 1. Demographic and clinical characteristics for included and excluded subjects/scans.**

*Abbreviation: Attention deficit/hyperactivity disorder; IQ, intelligence quotient; RMS, root‐mean‐square; SES, socioeconomic status.*

*^1^Excluding specific phobias.*

*^2^Community diagnosed.*

**
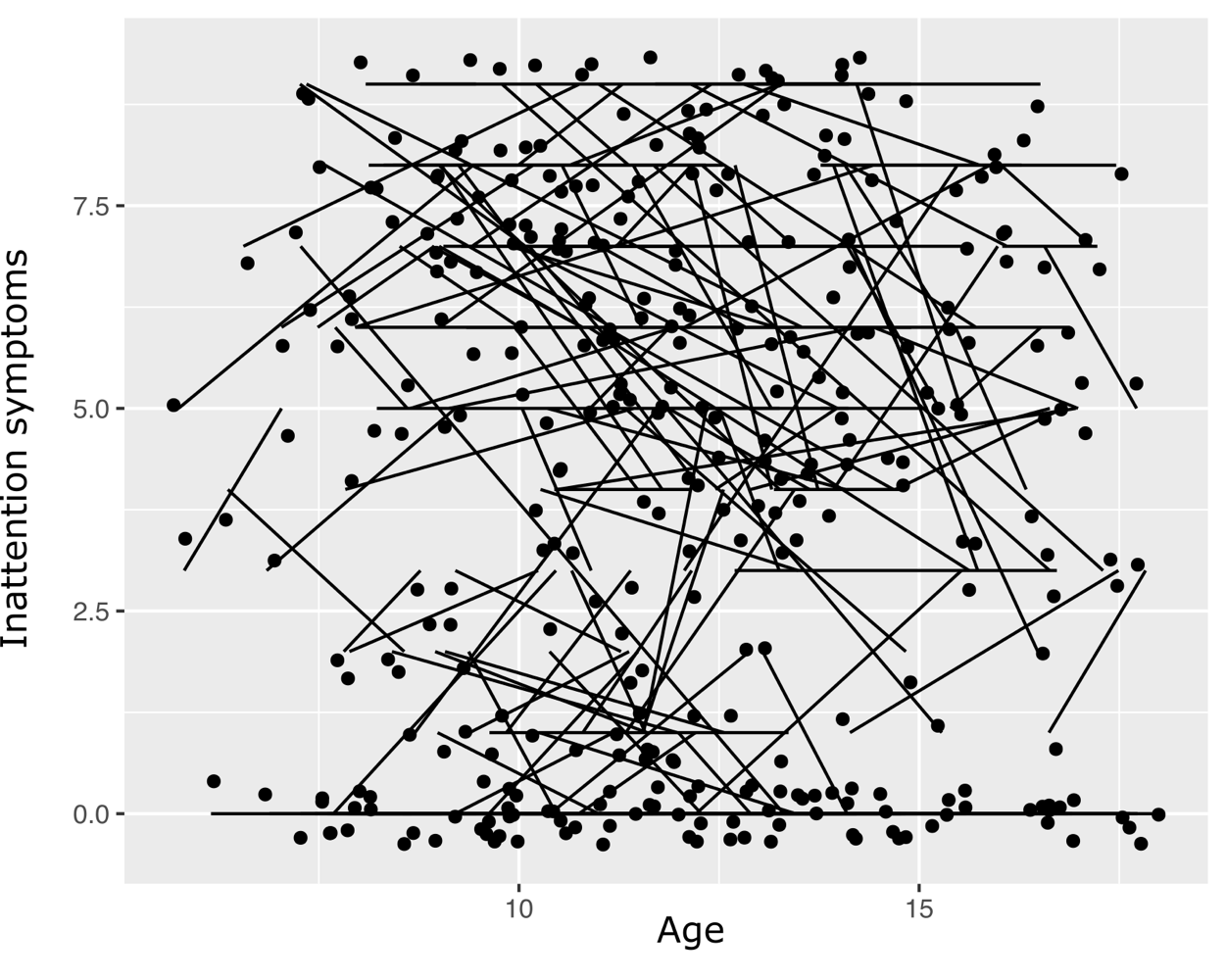
**

**Supplementary Figure 1.** Plot summarizing change in symptoms of inattention over time in N=167 children and adolescents. Each datapoint represents a symptom assessment, while lines represent within-subject trajectories of symptom change over time. A “jitter” was included to improve visibility of individual datapoints.

**
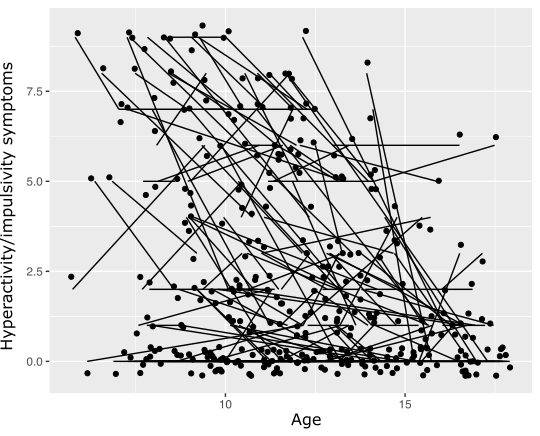
**

**Supplementary Figure 2.** Plot summarizing change in symptoms of hyperactivity/impulsivity over time in N=167 children and adolescents. Each datapoint represents a symptom assessment, while lines represent within-subject trajectories of symptom change over time. A “jitter” was included to improve visibility of individual datapoints.

**References**

1. Association AP. Diagnostic and statistical manual of mental disorders (DSM-5®). American Psychiatric Pub; 2013.

2. Fayyad J, De Graaf R, Kessler R, Alonso J, Angermeyer M, Demyttenaere K, et al. Cross-national prevalence and correlates of adult attention-deficit hyperactivity disorder. Br J Psychiatry. 2007;190:402–409.

3. Polanczyk GV, Salum GA, Sugaya LS, Caye A, Rohde LA. Annual research review: A meta-analysis of the worldwide prevalence of mental disorders in children and adolescents. J Child Psychol Psychiatry. 2015;56:345–365.

4. Franke B, Michelini G, Asherson P, Banaschewski T, Bilbow A, Buitelaar JK, et al. Live fast, die young? A review on the developmental trajectories of ADHD across the lifespan. Eur Neuropsychopharmacol. 2018;28:1059–1088.

5. Pingault J-B, Viding E, Galéra C, Greven CU, Zheng Y, Plomin R, et al. Genetic and Environmental Influences on the Developmental Course of Attention-Deficit/Hyperactivity Disorder Symptoms From Childhood to Adolescence. JAMA Psychiatry. 2015;72:651–658.

6. Larsson H, Dilshad R, Lichtenstein P, Barker ED. Developmental trajectories of DSM-IV symptoms of attention-deficit/hyperactivity disorder: genetic effects, family risk and associated psychopathology. J Child Psychol Psychiatry. 2011;52:954–963.

7. Bozhilova NS, Michelini G, Kuntsi J, Asherson P. Mind wandering perspective on attention-deficit/hyperactivity disorder. Neurosci Biobehav Rev. 2018;92:464–476.

8. Castellanos FX, Proal E. Large-scale brain systems in ADHD: beyond the prefrontal–striatal model. Trends in Cognitive Sciences. 2012;16:17–26.

9. Sonuga-Barke EJS, Castellanos FX. Spontaneous attentional fluctuations in impaired states and pathological conditions: a neurobiological hypothesis. Neurosci Biobehav Rev. 2007;31:977–986.

10. Konrad K, Di Martino A, Aoki Y. Brain volumes and intrinsic brain connectivity in ADHD. Oxford Textbook of Attention Deficit Hyperactivity Disorder. 2018:57.

11. Di Martino A, Zuo X-N, Kelly C, Grzadzinski R, Mennes M, Schvarcz A, et al. Shared and distinct intrinsic functional network centrality in autism and attention-deficit/hyperactivity disorder. Biol Psychiatry. 2013;74:623–632.

12. Cubillo A, Halari R, Ecker C, Giampietro V, Taylor E, Rubia K. Reduced activation and inter-regional functional connectivity of fronto-striatal networks in adults with childhood Attention-Deficit Hyperactivity Disorder (ADHD) and persisting symptoms during tasks of motor inhibition and cognitive switching. J Psychiatr Res. 2010;44:629–639.

13. McCarthy H, Skokauskas N, Mulligan A, Donohoe G, Mullins D, Kelly J, et al. Attention network hypoconnectivity with default and affective network hyperconnectivity in adults diagnosed with attention-deficit/hyperactivity disorder in childhood. JAMA Psychiatry. 2013;70:1329–1337.

14. Sudre G, Choudhuri S, Szekely E, Bonner T, Goduni E, Sharp W, et al. Estimating the Heritability of Structural and Functional Brain Connectivity in Families Affected by Attention-Deficit/Hyperactivity Disorder. JAMA Psychiatry. 2017;74:76–84.

15. Uddin LQ, Kelly AMC, Biswal BB, Margulies DS, Shehzad Z, Shaw D, et al. Network homogeneity reveals decreased integrity of default-mode network in ADHD. J Neurosci Methods. 2008;169:249–254.

16. Kessler D, Angstadt M, Welsh RC, Sripada C. Modality-spanning deficits in attention-deficit/hyperactivity disorder in functional networks, gray matter, and white matter. J Neurosci. 2014;34:16555–16566.

17. Cai W, Griffiths K, Korgaonkar MS, Williams LM, Menon V. Inhibition-related modulation of salience and frontoparietal networks predicts cognitive control ability and inattention symptoms in children with ADHD. Mol Psychiatry. 2019. 29 October 2019. https://doi.org/10.1038/s41380-019-0564-4.

18. van Rooij D, Hartman CA, Mennes M, Oosterlaan J, Franke B, Rommelse N, et al. Altered neural connectivity during response inhibition in adolescents with attention-deficit/hyperactivity disorder and their unaffected siblings. Neuroimage Clin. 2015;7:325–335.

19. Lukito S, Norman L, Carlisi C, Radua J, Hart H, Simonoff E, et al. Comparative meta-analyses of brain structural and functional abnormalities during cognitive control in attention-deficit/hyperactivity disorder and autism spectrum disorder. Psychol Med. 2020:1–26.

20. Norman LJ, Carlisi C, Lukito S, Hart H, Mataix-Cols D, Radua J, et al. Structural and functional brain abnormalities in attention-deficit/hyperactivity disorder and obsessive-compulsive disorder: a comparative meta-analysis. JAMA Psychiatry. 2016;73:815–825.

21. Gottesman II, Gould TD. The endophenotype concept in psychiatry: etymology and strategic intentions. Am J Psychiatry. 2003;160:636–645.

22. van Lieshout M, Luman M, Schweren LJS, Twisk JWR, Faraone SV, Heslenfeld DJ, et al. The Course of Neurocognitive Functioning and Prediction of Behavioral Outcome of ADHD Affected and Unaffected Siblings. J Abnorm Child Psychol. 2019;47:405–419.

23. Kendler KS, Neale MC. Endophenotype: a conceptual analysis. Mol Psychiatry. 2010;15:789–797.

24. Rubia K. Cognitive Neuroscience of Attention Deficit Hyperactivity Disorder (ADHD) and Its Clinical Translation. Front Hum Neurosci. 2018;12:100.

25. Rosseel Y. lavaan: An R Package for Structural Equation Modeling. Journal of Statistical Software. 2012;48:1–36.

26. Lohmann G, Margulies DS, Horstmann A, Pleger B, Lepsien J, Goldhahn D, et al. Eigenvector centrality mapping for analyzing connectivity patterns in fMRI data of the human brain. PLoS One. 2010;5:e10232.

27. Wink AM, de Munck JC, van der Werf YD, van den Heuvel OA, Barkhof F. Fast eigenvector centrality mapping of voxel-wise connectivity in functional magnetic resonance imaging: implementation, validation, and interpretation. Brain Connectivity. 2012;2:265–274.

28. Zuo X-N, Ehmke R, Mennes M, Imperati D, Castellanos FX, Sporns O, et al. Network centrality in the human functional connectome. Cereb Cortex. 2012;22:1862–1875.

29. Kolskår KK, Alnæs D, Kaufmann T, Richard G, Sanders A-M, Ulrichsen KM, et al. Key Brain Network Nodes Show Differential Cognitive Relevance and Developmental Trajectories during Childhood and Adolescence. ENeuro. 2018;5.

30. Sato JR, Salum GA, Gadelha A, Vieira G, Zugman A, Picon FA, et al. Decreased centrality of subcortical regions during the transition to adolescence: A functional connectivity study. NeuroImage. 2015;104:44–51.

31. Antonenko D, Nierhaus T, Meinzer M, Prehn K, Thielscher A, Ittermann B, et al. Age-dependent effects of brain stimulation on network centrality. NeuroImage. 2018;176:71–82.

32. Karim HT, Andreescu C, Tudorascu D, Smagula SF, Butters MA, Karp JF, et al. Intrinsic functional connectivity in late-life depression: trajectories over the course of pharmacotherapy in remitters and non-remitters. Mol Psychiatry. 2017;22:450–457.

33. Anticevic A, Hu S, Zhang S, Savic A, Billingslea E, Wasylink S, et al. Global Resting-State Functional Magnetic Resonance Imaging Analysis Identifies Frontal Cortex, Striatal, and Cerebellar Dysconnectivity in Obsessive-Compulsive Disorder. Biological Psychiatry. 2014;75:595–605.

34. Gili T, Saxena N, Diukova A, Murphy K, Hall JE, Wise RG. The thalamus and brainstem act as key hubs in alterations of human brain network connectivity induced by mild propofol sedation. J Neurosci. 2013;33:4024–4031.

35. Abdallah CG, Averill LA, Collins KA, Geha P, Schwartz J, Averill C, et al. Ketamine Treatment and Global Brain Connectivity in Major Depression. Neuropsychopharmacology. 2017;42:1210–1219.

36. Driesen NR, McCarthy G, Bhagwagar Z, Bloch M, Calhoun V, D’Souza DC, et al. Relationship of resting brain hyperconnectivity and schizophrenia-like symptoms produced by the NMDA receptor antagonist ketamine in humans. Molecular Psychiatry. 2013;18:1199–1204.

37. Shaw P, Gilliam M, Liverpool M, Weddle C, Malek M, Sharp W, et al. Cortical development in typically developing children with symptoms of hyperactivity and impulsivity: support for a dimensional view of attention deficit hyperactivity disorder. American Journal of Psychiatry. 2011;168:143–151.

38. Hoogman M, Muetzel R, Guimaraes JP, Shumskaya E, Mennes M, Zwiers MP, et al. Brain imaging of the cortex in ADHD: a coordinated analysis of large-scale clinical and population-based samples. American Journal of Psychiatry. 2019;176:531–542.

39. Salum GA, Sonuga-Barke E, Sergeant J, Vandekerckhove J, Gadelha A, Moriyama TS, et al. Mechanisms underpinning inattention and hyperactivity: neurocognitive support for ADHD dimensionality. Psychological Medicine. 2014;44:3189–3201.

40. Kolodny T, Mevorach C, Stern P, Biderman N, Ankaoua M, Tsafrir S, et al. Fronto-parietal engagement in response inhibition is inversely scaled with attention-deficit/hyperactivity disorder symptom severity. NeuroImage: Clinical. 2020;25:102119.

41. McLeod KR, Langevin LM, Goodyear BG, Dewey D. Functional connectivity of neural motor networks is disrupted in children with developmental coordination disorder and attention-deficit/hyperactivity disorder. Neuroimage Clin. 2014;4:566–575.

42. Muster R, Choudhury S, Sharp W, Kasparek S, Sudre G, Shaw P. Mapping the neuroanatomic substrates of cognition in familial attention deficit hyperactivity disorder. Psychol Med. 2019;49:590–597.

43. Sudre G, Frederick J, Sharp W, Ishii-Takahashi A, Mangalmurti A, Choudhury S, et al. Mapping associations between polygenic risks for childhood neuropsychiatric disorders, symptoms of attention deficit hyperactivity disorder, cognition, and the brain. Mol Psychiatry. 2019:10.1038/s41380-019-0350–0353.

44. Reich W. Diagnostic interview for children and adolescents (DICA). Journal of the American Academy of Child & Adolescent Psychiatry. 2000;39:59–66.

45. Wechsler D. WASI-II: Wechsler abbreviated scale of intelligence. PsychCorp; 2011.

46. Wechsler D. Wechsler preschool and primary scale of intelligence—fourth edition. The Psychological Corporation San Antonio, TX. 2012. 2012.

47. Ciric R, Rosen AFG, Erus G, Cieslak M, Adebimpe A, Cook PA, et al. Mitigating head motion artifact in functional connectivity MRI. Nat Protoc. 2018;13:2801–2826.

48. Gur RE, Moore TM, Rosen AFG, Barzilay R, Roalf DR, Calkins ME, et al. Burden of Environmental Adversity Associated With Psychopathology, Maturation, and Brain Behavior Parameters in Youths. JAMA Psychiatry. 2019;76:966–975.

49. Cui Z, Li H, Xia CH, Larsen B, Adebimpe A, Baum GL, et al. Individual variation in functional topography of association networks in youth. Neuron. 2020. 2020.

50. Gu S, Xia CH, Ciric R, Moore TM, Gur RC, Gur RE, et al. Unifying the Notions of Modularity and Core–Periphery Structure in Functional Brain Networks during Youth. Cerebral Cortex. 2020;30:1087–1102.

51. Ciric R, Wolf DH, Power JD, Roalf DR, Baum GL, Ruparel K, et al. Benchmarking of participant-level confound regression strategies for the control of motion artifact in studies of functional connectivity. Neuroimage. 2017;154:174–187.

52. Esteban O, Markiewicz CJ, Blair RW, Moodie CA, Isik AI, Erramuzpe A, et al. fMRIPrep: a robust preprocessing pipeline for functional MRI. Nature Methods. 2019;16:111–116.

53. Lohmann G, Loktyushin A, Stelzer J, Scheffler K. Eigenvector centrality mapping for ultrahigh resolution fMRI data of the human brain. Biorxiv. 2018:494732.

54. Zeileis A, Kleiber C, Jackman S. Regression models for count data in R. Journal of Statistical Software. 2008;27:1–25.

55. Jackman S. pscl: Classes and Methods for R Developed in the Political Science Computational Laboratory. United States Studies Centre, University of Sydney, Sydney, New South Wales, Australia; 2020.

56. Worsley K. Random field theory. Statistical Parametric Mapping: The Analysis of Functional Brain Images. 2011:232–245.

57. Eklund A, Nichols TE, Knutsson H. Cluster failure: Why fMRI inferences for spatial extent have inflated false-positive rates. Proceedings of the National Academy of Sciences. 2016;113:7900–7905.

58. Cox RW. AFNI: software for analysis and visualization of functional magnetic resonance neuroimages. Computers and Biomedical Research. 1996;29:162–173.

59. van Rooij D, Hoekstra PJ, Mennes M, von Rhein D, Thissen AJAM, Heslenfeld D, et al. Distinguishing Adolescents With ADHD From Their Unaffected Siblings and Healthy Comparison Subjects by Neural Activation Patterns During Response Inhibition. Am J Psychiatry. 2015;172:674–683.

60. Norman LJ, Taylor SF, Liu Y, Radua J, Chye Y, De Wit SJ, et al. Error Processing and Inhibitory Control in Obsessive-Compulsive Disorder: A Meta-analysis Using Statistical Parametric Maps. Biol Psychiatry. 2019;85:713–725.

61. Rubia K, Halari R, Cubillo A, Mohammad A-M, Brammer M, Taylor E. Methylphenidate normalises activation and functional connectivity deficits in attention and motivation networks in medication-naive children with ADHD during a rewarded continuous performance task. Neuropharmacology. 2009;57:640–652.

62. Vloet TD, Gilsbach S, Neufang S, Fink GR, Herpertz-Dahlmann B, Konrad K. Neural mechanisms of interference control and time discrimination in attention-deficit/hyperactivity disorder. J Am Acad Child Adolesc Psychiatry. 2010;49:356–367.

63. Wolf RC, Plichta MM, Sambataro F, Fallgatter AJ, Jacob C, Lesch K-P, et al. Regional brain activation changes and abnormal functional connectivity of the ventrolateral prefrontal cortex during working memory processing in adults with attention-deficit/hyperactivity disorder. Hum Brain Mapp. 2009;30:2252–2266.

64. Cao Q, Zang Y, Sun L, Sui M, Long X, Zou Q, et al. Abnormal neural activity in children with attention deficit hyperactivity disorder: a resting-state functional magnetic resonance imaging study. Neuroreport. 2006;17:1033–1036.

65. Szekely E, Sudre GP, Sharp W, Leibenluft E, Shaw P. Defining the Neural Substrate of the Adult Outcome of Childhood ADHD: A Multimodal Neuroimaging Study of Response Inhibition. Am J Psychiatry. 2017;174:867–876.

66. Hart H, Chantiluke K, Cubillo AI, Smith AB, Simmons A, Brammer MJ, et al. Pattern classification of response inhibition in ADHD: toward the development of neurobiological markers for ADHD. Hum Brain Mapp. 2014;35:3083–3094.

67. Rubia K, Smith AB, Halari R, Matsukura F, Mohammad M, Taylor E, et al. Disorder-specific dissociation of orbitofrontal dysfunction in boys with pure conduct disorder during reward and ventrolateral prefrontal dysfunction in boys with pure ADHD during sustained attention. Am J Psychiatry. 2009;166:83–94.

68. Passarotti AM, Sweeney JA, Pavuluri MN. Neural correlates of response inhibition in pediatric bipolar disorder and attention deficit hyperactivity disorder. Psychiatry Res. 2010;181:36–43.

69. Cubillo A, Halari R, Giampietro V, Taylor E, Rubia K. Fronto-striatal underactivation during interference inhibition and attention allocation in grown up children with attention deficit/hyperactivity disorder and persistent symptoms. Psychiatry Research: Neuroimaging. 2011;193:17–27.

70. Cortese S, Kelly C, Chabernaud C, Proal E, Di Martino A, Milham MP, et al. Toward systems neuroscience of ADHD: a meta-analysis of 55 fMRI studies. American Journal of Psychiatry. 2012;169:1038–1055.

71. Rubia K, Alegria AA, Cubillo AI, Smith AB, Brammer MJ, Radua J. Effects of stimulants on brain function in attention-deficit/hyperactivity disorder: a systematic review and meta-analysis. Biol Psychiatry. 2014;76:616–628.

72. Westwood S, Asherson P, Kadosh RC, Wexler B, Rubia K. A novel neurotherapy of transcranial direct current stimulation (tDCS) combined with cognitive training in ADHD children. Brain Stimulation: Basic, Translational, and Clinical Research in Neuromodulation. 2019;12:521.

73. Alegria AA, Wulff M, Brinson H, Barker GJ, Norman LJ, Brandeis D, et al. Real-time fMRI neurofeedback in adolescents with attention deficit hyperactivity disorder. Hum Brain Mapp. 2017;38:3190–3209.

74. Schulz KP, Newcorn JH, Fan JIN, Tang CY, Halperin JM. Brain activation gradients in ventrolateral prefrontal cortex related to persistence of ADHD in adolescent boys. Journal of the American Academy of Child & Adolescent Psychiatry. 2005;44:47–54.

75. Shaw P, Sudre G. Adolescent attention deficit hyperactivity disorder: understanding teenage symptom trajectories. Biological Psychiatry. 2020. 2020.

76. Swanson JM, Schuck S, Porter MM, Carlson C, Hartman CA, Sergeant JA, et al. Categorical and dimensional definitions and evaluations of symptoms of ADHD: history of the SNAP and the SWAN rating scales. The International Journal of Educational and Psychological Assessment. 2012;10:51.

77. Gorgolewski K, Burns CD, Madison C, Clark D, Halchenko YO, Waskom ML, et al. Nipype: a flexible, lightweight and extensible neuroimaging data processing framework in python. Frontiers in Neuroinformatics. 2011;5:13.

78. Tustison NJ, Avants BB, Cook PA, Zheng Y, Egan A, Yushkevich PA, et al. N4ITK: improved N3 bias correction. IEEE Transactions on Medical Imaging. 2010;29:1310–1320.

79. Avants BB, Epstein CL, Grossman M, Gee JC. Symmetric diffeomorphic image registration with cross-correlation: evaluating automated labeling of elderly and neurodegenerative brain. Med Image Anal. 2008;12:26–41.

80. Zhang Y, Brady M, Smith S. Segmentation of brain MR images through a hidden Markov random field model and the expectation-maximization algorithm. IEEE Transactions on Medical Imaging. 2001;20:45–57.

81. Jenkinson M, Bannister P, Brady M, Smith S. Improved optimization for the robust and accurate linear registration and motion correction of brain images. Neuroimage. 2002;17:825–841.

82. Greve DN, Fischl B. Accurate and robust brain image alignment using boundary-based registration. Neuroimage. 2009;48:63–72.

83. Hallquist MN, Hwang K, Luna B. The nuisance of nuisance regression: spectral misspecification in a common approach to resting-state fMRI preprocessing reintroduces noise and obscures functional connectivity. Neuroimage. 2013;82:208–225.

84. Smith SM, Brady JM. SUSAN—a new approach to low level image processing. International Journal of Computer Vision. 1997;23:45–78.

85. Tzourio-Mazoyer N, Landeau B, Papathanassiou D, Crivello F, Etard O, Delcroix N, et al. Automated anatomical labeling of activations in SPM using a macroscopic anatomical parcellation of the MNI MRI single-subject brain. Neuroimage. 2002;15:273–289.

86. Power JD. A simple but useful way to assess fMRI scan qualities. Neuroimage. 2017;154:150–158.

87. Bolt T, Nomi JS, Rubinov M, Uddin LQ. Correspondence between evoked and intrinsic functional brain network configurations. Hum Brain Mapp. 2017;38:1992–2007.
